# Investigating Microstructural Abnormalities and Neurocognition in Sub-Acute and Chronic Traumatic Brain Injury Patients with Normal-Appearing White Matter: A Preliminary Diffusion Tensor Imaging Study

**DOI:** 10.3389/fneur.2017.00097

**Published:** 2017-03-20

**Authors:** Eyesha Hashim, Eduardo Caverzasi, Nico Papinutto, Caroline E. Lewis, Ruiwei Jing, Onella Charles, Shudong Zhang, Amy Lin, Simon J. Graham, Tom A. Schweizer, Aditya Bharatha, Michael D. Cusimano

**Affiliations:** ^1^Department of Neurosurgery, St. Michael’s Hospital, Toronto, ON, Canada; ^2^Department of Neurology, University of California at San Francisco, San Francisco, CA, USA; ^3^Department of Brain and Behavioral Sciences, University of Pavia, Italy; ^4^Department of Radiology, St. Michael’s Hospital, Toronto, ON, Canada; ^5^Sunnybrook Research Institute, University of Toronto, Toronto, ON, Canada; ^6^Department of Medical Imaging, St. Michael’s Hospital, University of Toronto, Toronto, ON, Canada; ^7^Department of Medical Imaging and Neurosurgery at the University of Toronto, Toronto, ON, Canada; ^8^Faculty of Medicine, Department of Surgery, Division of Neurosurgery, University of Toronto, Toronto, ON, Canada

**Keywords:** diffusion tensor imaging, chronic traumatic brain injury, white matter microstructure, human, voxel-based analysis, sub-acute traumatic brain injury, normal-appearing white matter

## Abstract

For a significant percentage of subjects, with chronic traumatic brain injury (TBI), who report persisting cognitive impairment and functional loss, the diagnosis is often impeded by the fact that routine neuroimaging often does not reveal any abnormalities. In this paper, we used diffusion tensor imaging (DTI) to investigate the apparently normal white matter (as assessed by routine magnetic resonance imaging) in the brains of 19 subjects with sub-acute (9) and chronic (10) TBI. We also assessed memory, executive function, and visual-motor coordination in these subjects. Using a voxel-wise approach, we investigated if parameters of diffusion were significantly different between TBI subjects and 17 healthy controls (HC), who were demographically matched to the TBI group. We also investigated if changes in DTI parameters were associated with neuropsychological performance in either group. Our results indicate significantly increased mean and axial diffusivity (MD and AD, respectively) values in widespread brain locations in TBI subjects, while controlling for age, sex, and time since injury. HC performed significantly better than the TBI subjects on tests of memory and executive function, indicating the persisting functional loss in chronic TBI. We found no correlation between diffusion parameters and performance on test of executive function in either group. We found negative correlation between FA and composite memory scores, and positive correlation between RD and visuomotor coordination test scores, in various tracts in both groups. Our study suggests that changes in MD and AD can indicate persisting micro-structure abnormalities in normal-appearing white matter in the brains of subjects with chronic TBI. Our results also suggest that FA in major white matter tracts is correlated with memory in health and in disease, alike; larger and longitudinal studies are needed to discern potential differences in these correlations in the two groups.

## Introduction

Traumatic brain injury (TBI) often results in cognitive and functional deficits, impairs daily life functioning, and degrades the quality of life of the injured person significantly in the acute stage (1). Furthermore, the cognitive and functional deficits may persist and yet remain untreated in the chronic stage, particularly when routine diagnostic imaging [e.g., structural magnetic resonance imaging (MRI), and computed tomography (CT)] does not reveal any gross structural abnormalities, indicative of the cognitive or functional loss, in the brain of the injured patients. A large literature indicates that these persisting deficits are linked to microstructural damage in the brain (2). Diffusion tensor imaging (DTI) (3), which uses the diffusion of water molecules to assess microstructure in both pathological and normal brain tissue, has frequently been used in the past decade to investigate microstructural damage in patients with TBI (4–6) [also see the review by Hulkower et al. (7)]. The DTI studies of TBI often investigate the change in one or more of the classic diffusion parameters—axial diffusivity (AD), radial diffusivity (RD), mean diffusivity (MD), and fractional anisotropy (FA)—using either region of interest (ROI) (8, 9) or whole brain voxel-wise (10, 11) approaches. Most of these studies have focused on FA alone (10) or FA and MD (12, 13), the two parameters that describe the overall or average diffusion trends. These studies suggest a general trend of decrease in directionality of diffusion as measured by FA (7, 10, 14, 15) and an increase in the overall diffusion as measured by MD (7, 16, 17) in subjects with chronic TBI and associate these changes with diffuse axonal injury (DAI).

The DAI, an example of microstructural disruption resulting from TBI, is thought to be one of the major causes of neurocognitive deficits after TBI (18, 19). However, given the complexity of the various cognitive and executive function networks in the brain, it is important to investigate the role of DAI in neurocognitive deficits after TBI in situations where no other confounding structural anomalies (e.g., focal lesions indicative of the cognitive or functional loss) are present and by adopting a whole-brain analysis approach. The DTI is an excellent technique to investigate the DAI in TBI, however, with notable exceptions (20, 21), most of the DTI studies investigating the correlation between neurocognitive function and DTI parameters did not exclude subjects with white matter abnormalities as assessed with routine diagnostic imaging (22–25). Furthermore, many of these DTI studies investigating neurocognitive function after TBI are restricted only to FA and MD and only to TBI patients, excluding healthy controls (HC) from the correlation analysis (9, 22, 23, 26–28). A few studies investigated this correlation in all subjects including HC using an ROI approach (22, 23, 29, 30) and only a fraction of these extended the correlation analysis to whole brain white matter tracts using the voxel-wise approach (31–33). While the aforementioned studies present similar conditions, these studies either did not investigate the presence of gross or focal white matter abnormalities (31, 33) or did not exclude the subjects with such abnormalities from the extended investigation of neurocognitive deficits. The present study builds on these latter studies of the whole brain white matter tracts using a voxel-wise analysis approach to elucidate the relationship between neuro-cognition and white matter microstructure in sub-acute and chronic TBI patients with normal-appearing white matter (NAWM).

In this pilot study, we investigate if the classic DTI parameters (FA, MD, RD and AD) exhibit any changes in patients with sub-acute and chronic TBI of all severities but with no gross abnormalities on routine MRI. We also aim to explore if the relationship between the classic DTI parameters and neurocognitive function is altered when compared with HC. This knowledge is essential as part of considering whether classic DTI parameters are useful biomarkers of the degree of neurocognitive deficit after TBI.

## Materials and Methods

### Participants

This study was performed at St. Michael’s Hospital in Toronto, ON, Canada with approval from the hospital’s Research Ethics Board. Patients with a positive history of TBI were recruited from the Trauma/Neurosurgery Ward, Neurosurgery and Head Injury Clinics, and Trauma Registry Centre at the hospital. Inclusion criteria for TBI patients required that they were within 16–70 years of age and had sustained an isolated, closed head TBI with Glasgow Coma Score [GCS; (34)] in the range 5–15 as assessed within 12 h of injury. The HC were recruited through the word-of-mouth and through online advertisement. For HC, the inclusion criterion was to be within 16–70 years of age, while the exclusion criterion included having a history of TBI, motor vehicle collisions, or falls. The exclusion criteria, as applicable to both groups, included the presence of non-hemorrhagic or micro-hemorrhagic brain lesions as assessed by fluid-attenuated inversion recovery (FLAIR) and gradient recalled echo (GRE) MRI. The presence of small superficial contusions and superficial hemosiderin staining was not considered to be an exclusion criterion, as these lesions are not expected to affect the central white matter tracts that this study aimed to analyze. The exclusion criterion for both groups also included incomplete assessment of neuro-cognition as tested with standardized neuropsychological (NP) tests (see below for more details). Subjects with existing substance abuse were excluded from the study, as this dependency can potentially alter cognition (35), and is known to be associated with microstructural abnormalities with altered FA values (36). Both groups also were subject to standard MRI exclusion criteria (e.g., claustrophobia, ferromagnetic implants) and were required to possessed adequate verbal English language skills. All participants provided informed consent to participate in the study, and received monetary compensation for the time spent on the study.

### NP Testing

Cognitive function was assessed in all subjects in the three categories of memory, executive function, and visual-motor coordination. These assessments were performed using the following standardized NP tests. Memory was assessed with the Digit Span subtest (37) of the Wechsler Adult Intelligence Scale (WAIS-III) (38) and the Hopkins Verbal Learning Test (HVLT) (39). Executive function was assessed using the Token Test (40), the Phonemic Fluency subtest, which is a part of the Neurosensory Center Comprehensive Examination for Aphasia (41), and the Digit Symbol subtest of the Wechsler Adult Intelligence Scale (WAIS-III) (38). The visuomotor coordination skill was assessed using the Grooved Pegboard test (42). The National Adult Reading Test (NART) (43) was used as a measure of verbal IQ. Except for the Grooved Pegboard, all tests were administered using pen and paper.

In the Digit Span test, the subjects were asked to recall numbers in forward and backward order and the number of correct responses was recorded in each case. The HVLT consisted of three trials; the total correct responses, incorrect responses, and intrusions were recorded for each trial and the number of correct delayed recall responses at the end of the three trials was also recorded. For the Token test, the total correct responses in each of the two trials performed were recorded. The Phonemic Fluency test required subjects to list words starting with each of the letters F, A, and S; total correct responses, incorrect/repeated responses, and intrusions were recorded in each case. For the Digit Symbol test, the total number of correctly completed, incorrectly completed, skipped, and incomplete boxes were recorded. For the Grooved Pegboard test, the time (in seconds) taken to put all the pegs in the board and the number of pegs dropped during the procedure were noted. Subjects completed the task first with their dominant hand, then the non-dominant hand. The test thus resulted in four scores: time to complete with the dominant hand (dominant time), the number of drops with the dominant hand (dominant drop), time to complete with the non-dominant hand (non-dominant time), and the number of drops with the non-dominant hand (non-dominant drop).

For each of the NP tests administered, a higher score indicates better performance. The one exception is the Grooved Pegboard test, in which a higher score indicates poorer visuomotor coordination.

### MRI Protocol

Imaging was performed with a research-dedicated MRI system at 3.0 T (MR750, GE Healthcare, Waukesha, WI, USA) at Sunnybrook Health Sciences Centre in Toronto. The MRI protocol for each subject (TBI patients and controls) included a three-dimensional (3D) longitudinal relaxation time (T_1_)-weighted sequence to rule out gross structural abnormalities and for anatomical reference and two-dimensional (2D) FLAIR and GRE sequences to detect microbleeds, along with a 2D DTI sequence. The parameters for these MRI sequences were as follows: 3D axial T_1_-weighted inversion-recovery prepped fast gradient echo imaging—in-plane resolution = 0.9 mm × 0.9 mm; field of view (FOV) = 220 mm × 165 mm; slice thickness = 1.4 mm; flip angle = 15°; repetition time (TR) = 8.2 ms; echo time (TE) = 3.2 ms, axial FLAIR imaging—resolution = 0.9 mm × 1.1 mm × 3.0 mm; FOV = 220 mm × 200 mm; flip angle = 90°; TR = 9,950 ms; TE = 96 ms; axial GRE imaging—resolution = 0.8 mm × 1.0 mm × 3.0 mm; FOV = 200 mm × 200 mm; flip angle = 20°; TR = 784 ms; TE = 35 ms; and axial DTI imaging—FOV = 240 mm × 240 mm; resolution = 1.9 mm × 1.9 mm; slice thickness = 3 mm; non-collinear diffusion directions = 11 with *b* = 1,000 s/mm^2^ and with two volumes applied without diffusion sensitizing gradients (*b* = 0 s/mm^2^), averages = 4; TR = 9,400 ms; TE = 89.7 ms. The MRI protocol lasted approximately 40 min.

### DTI Data Processing

Diffusion tensor imaging data were processed using the Oxford Centre for Functional MRI of the Brain (FMRIB) Software Library (FSL- version 5.0.5—http://www.fmrib.ox.ac.uk/fsl) (44). The diffusion data were corrected for distortions due to eddy currents and minor head motion using the *b* = 0 image as a reference. Brain extraction was then performed on these data using the FSL brain extraction tool (45). The diffusion tensor model was fitted to the images of the extracted brain tissue to produce maps of FA, MD, RD, and AD in individual subjects. Voxel-wise analyses of the DTI parameters were performed using the Tract-Based Spatial Statistics (TBSS) package (46). As part of TBSS processing, the FA image of each subject was transformed into the Montreal Neurological Institute (MNI)-152 standard space (47), using non-linear registration (44, 48) to the FMRIB58_FA_1mm image (average high-resolution FA image of 58 subjects in MNI 152 standard space). The mean FA maps of all subjects enrolled in the study were then created. A mean white matter skeleton was subsequently generated using a threshold of FA > 0.2 to include only the centers of white matter tracts common to the entire group. The FA data from each subject were then projected onto this mean FA skeleton to generate a 4D data matrix (*x, y, z*, FA) characterizing the group. The non-linear transformations used in this procedure were then applied to create analogous 4D data matrices of the skeletonized MD, RD, and AD values, respectively. These matrices were used for the inter-group statistical analyses.

### Statistical Analyses

A Student’s *t*-test was used to compare the mean NP test scores between the two groups, after assessing for normality of the data. Differences were considered statistically significant using a threshold *p-*value ≤0.05.

A General Linear Model (GLM) (49) was applied using a voxel-based approach to each of the 4D data matrices of FA, MD, AD, and RD within the FSL framework to investigate the relationship of DTI parameters with the group (TBI versus HC). The GLM was applied using non-parametric permutation-based statistics by employing the *randomize* tool in FSL with threshold-free cluster enhancement and 5,000 permutations (50). Age, sex, and time since injury were included as non-interacting covariates in the GLM. The contrasts were designed to investigate if the two groups had statistically significant differences in DTI parameters (TBI > HC and HC > TBI).

The investigation of whether DTI parameters were correlated with NP test performance was also performed with the *randomize* tool within the framework of GLM in FSL. For this analysis, a composite score for each category of NP function (memory, executive function, and visuomotor coordination) was calculated using the following procedure. First, the Pearson correlation coefficient (R) was calculated to assess the correlation between scores and sub-scores of various tests within the same category and only those subtests that had significant positive correlation with each other (*R* > 0.5, *p*-value ≤0.05) were included in the subsequent analyses. These selected tests/subtests scores for all subjects (TBI and HC) were then converted into ranks. Finally, a composite score for each category was calculated as the mean of ranks of all subtests and tests in that category. The correlation and rank calculations were performed using the Statistical Analysis System, 9.4 (SAS Institute Inc. 2013, Cary, NC, USA). These composites scores for NP tests were entered in the GLM design matrix for investigation of the correlation between DTI parameters and NP test performance. First, an investigation of whether the correlation between NP test performance and DTI parameters varied according to the group was performed. For this, an interaction term for each NP test category was added to the design matrix and contrasts were designed to determine positive and negative interactions between composite test scores in the three NP categories and group predictor variable. Afterward, correlations of the composite scores in the three NP categories were investigated with contrasts designed to calculate positive as well as negative correlations. For these analyses involving NP tests, IQ and education were also included in the GLM as non-interacting covariates.

A *p*-value ≤0.05, corrected for multiple comparisons within *randomize* by controlling for the family-wise error rate (51), was considered significant for all statistical comparisons performed within the framework of the GLM in FSL.

### Identifying the Regions with Significant Inter-Group Differences/Correlations

The *randomize* outputs consisting of *p*-values corrected for multiple comparisons were thresholded to keep voxels with *p*-value ≤0.05. The FSL atlas query and cluster identification tools (merged as the *autoaq* algorithm) were used on the thresholded images to identify various white matter tracts using the Johns Hopkins University White Matter Labels atlas (52). To eliminate noise due to averaging and normalization, tracts with <50 significantly different voxels were not considered as tracts exhibiting structural differences between the two groups and were excluded from the reported results.

### *Post Hoc* Analyses

The *post hoc* analyses, where mentioned, were performed using only those voxels that were found significant at *p* ≤ 0.05 level. The *post hoc* analyses were also performed within the framework of the GLM in FSL and the results were corrected for multiple comparisons within *randomize* by controlling for the family-wise error rate (51).

## Results

### Subjects

A total of 52 patients and 18 HC met the inclusion criteria and underwent MRI and standardized NP tests. FLAIR and GRE images of all the subjects were reviewed by two board-certified neuroradiologists to rule out non-hemorrhagic (53) and micro-hemorrhagic (54) lesions. Twelve patients were excluded from the study: five did not complete the MRI exam, four had significant head motion during MRI and three withdrew their consent after MRI. Twenty-one patients and one HC were excluded from the analyses due to gross abnormalities in the central cerebral white matter, visible on FLAIR or GRE images. Most of the TBI subjects had small superficial contusions and superficial hemosiderin staining but were not excluded, as these lesions are not expected to affect the central white matter tracts that this study aimed to analyze. Nineteen patients with a history of trauma from a motor vehicle collision (5 patients) or fall (14 patients) were thus included in the study. Based on the GCS scores at the time of emergency department arrival, 15 patients had mild TBI (reference GCS: 13–15), three patients had moderate TBI (reference GCS: 9–12) and one patient had severe TBI (reference GCS ≤8). Time elapsed between injury and MRI for the present study ranged from 0.2 to 5.1 years (Table 1). Nine subjects were in the sub-acute stage (2 weeks < time since injury < 1 year), whereas the rest of the subjects were in the chronic stage after TBI (time since injury > 1 year) (7).

**Table 1:** **Group demographic variables**.

	TBI (*N* = 19; 12 males)		Healthy controls (*N* = 17; 10 males)	
	Median	IQR	Median	IQR
Age (years)	47	22.5	42	31
Education (years)	15	2.5	15	3
Glasgow Coma Score	14	2	Not applicable	
Time since injury (years)	1	1.8		

*Median and interquartile range (IQR) for demographic variables of traumatic brain injury (TBI) patients and control subjects*.

The group of HC consisted of individuals recruited through online advertisement as well as friends and family members of patients who attended the injury, trauma, or neurosurgery clinics at the hospital. The group of HC was matched by age, sex, and education to the group of TBI patients. Table 1 lists the demographic details of all subjects in the study.

### NP Performance

Healthy controls performed better than the TBI group on all NP tests, based on score results. However, only the scores for Digit Span, Phonemic Fluency, the third trial of the HVLT, and Digit Symbol tests were significantly higher in HC at *p*-value ≤0.05. A significant positive correlation was found between the Digit Span scores (forward and backward), the total correct responses in each of the three trials of HVLT, and the correct delayed recall score in the memory category. In the executive function category, a significant positive correlation was found between the total correct phonemic score, the total correct score in the Digit Symbol test, and the total score in each of the two Token test trials. In the visuomotor coordination category, the dominant and non-dominant times were found to have a significant positive correlation. No significant difference was found in the verbal IQ of the two groups as measured with the NART. The results of NP test performance are summarized in Table 2.

**Table 2:** **NP performance results**.

NP test	TBI	Healthy controls	*p*-Value
		
	Median/interquartile range (IQR)	Median/IQR	
National Adult Reading Test (verbal IQ)	34/15	39.5/14	0.1
**Memory**			
Digit span (forward and backward)	9/4; 6/3.5	12/3; 7.5/4.8	0.003*; 0.03*
Hopkins Verbal Learning Test (3 trials and delayed recall)	6/3; 9/2.5; 8/4; 8/2.5	5.5/2.8; 8.5/3; 10/2; 8.5/3.8	0.8; 0.4; 0.04*; 0.2
**Executive function**			
Token test (2 trials)	21/3; 22/0	21/2; 22/1	0.2; 0.7
Digit symbol (total correct)	64/9	71.5/29.3	0.02*
Phonemic fluency (total correct)	36/12	42/13.5	0.02*
**Visuomotor coordination**			
Pegboard (dominant and non-dominant time- seconds)	70/11; 79/30.5	60/28.5; 70/20	0.16; 0.08

*List of subtests and tests in each category of neuropsychological (NP) function that were found to have significant positive correlation with each other. Higher values imply better performance on the corresponding NP test except for the Pegboard where the reverse is true. The last column lists the *p*-value for Student’s *t*-test*.

*represents statistically significant difference between the mean scores of the two groups.

### Inter-Group Differences in DTI Parameters

**Figure 1:**
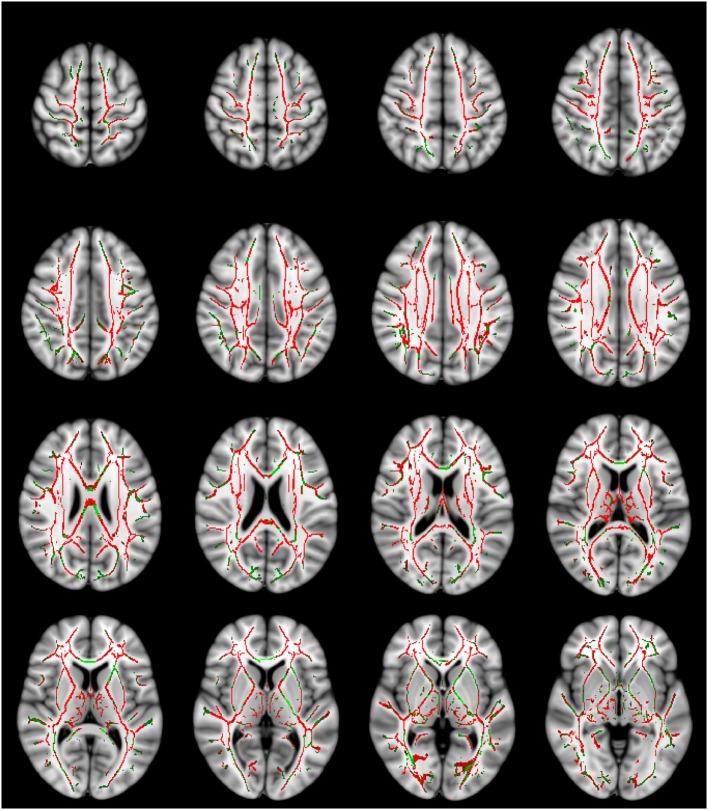
**Mean diffusivity (MD) values are significantly higher in the traumatic brain injury (TBI) group with normal-appearing white matter**. The MD contrast is overlaid on a standard Montreal Neurological Institute 152 T_1_ 1 mm brain and the mean fractional anisotropy skeleton (green—display threshold 0.2–0.8). The voxels where MD was found to be significantly higher in the TBI group (*p*-value ≤ 0.05) are shown in red. Every fourth transverse slice only is shown here to approximately cover the entire brain along the superior–inferior axis. Anatomical right side of the brain is shown on the left in this figure.

**Figure 2:**
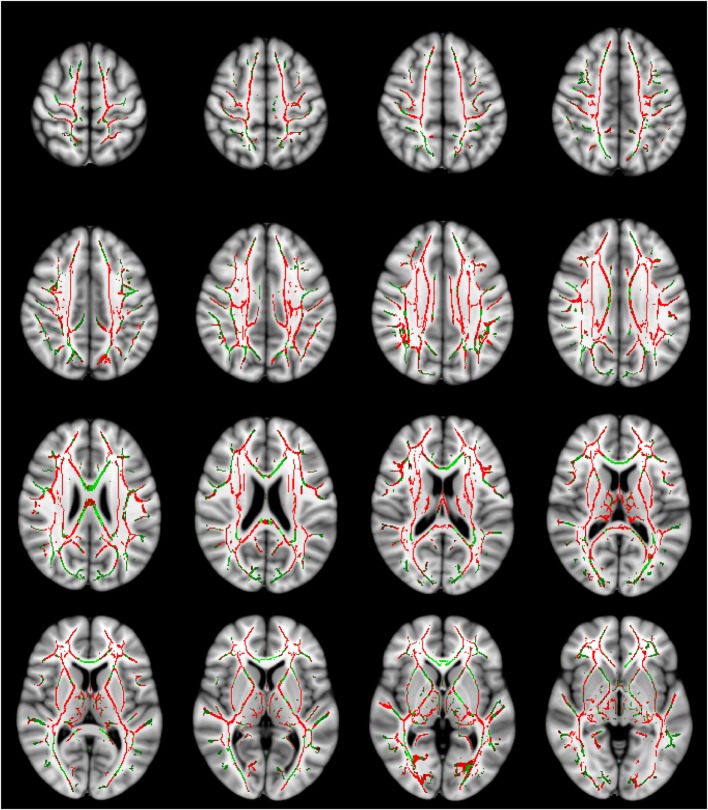
**Axial diffusivity (AD) values are significantly higher in the traumatic brain injury (TBI) group with normal-appearing white matter**. The AD contrast is overlaid on a standard Montreal Neurological Institute 152 T_1_ 1 mm brain and the mean fractional anisotropy skeleton (green—display threshold 0.2–0.8). The voxels where mean diffusivity was found to be significantly higher in the TBI group (*p*-value ≤ 0.05) are shown in red. Every fourth transverse slice only is shown here to approximately cover the entire brain along the superior–inferior axis. Anatomical right side of the brain is shown on the left in this figure.

Significantly increased MD and AD values were found in the TBI group compared to HC in several brain regions (controlling for age, sex, and time since injury). Table 3 lists MD and AD values in those tracts where significant inter-group differences in these variables were found. In particular, significant differences in MD and AD were found in the corpus callosum and bilateral internal capsule, external capsule, corona radiata, posterior thalamic radiations, and various other structures. Changes in AD were more widespread compared to those in MD (Figures 1 and 2). No significant differences between the two groups were found in FA and RD. Although trends of increased RD and FA in TBI were visible in several major white matter tracts, notable exceptions included a trend of decreased FA in TBI in the corpus callosum and the bilateral cingulum.

**Table 3:** **Tracts with significantly increased MD and AD values in TBI**.

Structure	MD			AD		
	Mean ± SD (×10^−4^ – mm^2^ s^−1^)		*p*-Value *(post hoc)*	Mean ± SD (×10^−3^ – mm^2^ s^−1^)		*p-*Value (*post hoc*)
	TBI	HC		TBI	HC	
Corpus callosum	8.1 ± 0.4	7.8 ± 0.3	0.03 (0.008)	1.5 ± 0.7	1.5 ± 0.5	0.03 (0.004)
Corticospinal tract-R	–	–	–	1.3 ± 0.7	1.2 ± 0.7	0.03 (0.005)
Corticospinal tract-L	–	–	–	1.2 ± 1	1.2 ± 0.5	0.04 (0.007)
Cerebral peduncle-R	6.8 ± 0.4	6.6 ± 0.4	0.04 (0.01)	1.5 ± 0.8	1.4 ± 0.6	0.03 (0.005)
Cerebral peduncle-L	–	–	–	1.4 ± 0.8	1.4 ± 0.8	0.03 (0.005)
Internal capsule-R	7.5 ± 0.3	7.2 ± 0.2	0.03 (0.006)	1.4 ± 0.6	1.3 ± 0.4	0.02 (0.003)
Internal capsule-L	7.4 ± 0.3	7.2 ± 0.2	0.04 (0.01)	1.3 ± 0.5	1.3 ± 0.3	0.02 (0.003)
External capsule-R	7.6 ± 0.3	7.3 ± 0.2	0.03 (0.009)	1.2 ± 0.6	1.1 ± 0.2	0.03 (0.003)
External capsule-L	7.4 ± 0.3	7.2 ± 0.2	0.04 (0.02)	1.2 ± 0.6	1.1 ± 0.3	0.02 (0.004)
Corona radiata-R	7.4 ± 0.3	7.2 ± 0.2	0.02 (0.003)	1.2 ± 0.5	1.2 ± 0.4	0.02 (0.002)
Corona radiata-L	7.4 ± 0.3	7.2 ± 0.2	0.03 (0.007)	1.2 ± 0.5	1.2 ± 0.4	0.02 (0.002)
Cingulum-R	7.8 ± 0.3	7.4 ± 0.2	0.03 (0.005)	1.2 ± 0.5	1.2 ± 0.3	0.03 (0.004)
Cingulum-L	7.7 ± 0.4	7.4 ± 0.2	0.03 (0.01)	1.2 ± 0.6	1.2 ± 0.4	0.03 (0.004)
Thalamic rad Post-R	8.3 ± 0.4	7.9 ± 0.4	0.03 (0.007)	1.5 ± 0.7	1.4 ± 0.5	0.03 (0.004)
Thalamic rad Post-L	8.0 ± 0.4	7.6 ± 0.4	0.04 (0.01)	1.4 ± 0.6	1.3 ± 0.5	0.04 (0.007)
Longitudinal fasc Sup-R	7.4 ± 0.3	7.1 ± 0.2	0.02 (0.006)	1.2 ± 0.6	1.1 ± 0.4	0.03 (0.004)
Longitudinal fasc Sup-L	7.5 ± 0.3	7.2 ± 0.2	0.03 (0.009)	1.2 ± 0.34	1.1 ± 0.3	0.03 (0.005)
Frontooccipital fasc Sup-R	7.1 ± 0.3	6.7 ± 0.2	0.02 (0.005)	1.1 ± 0.6	1.1 ± 0.5	0.02 (0.003)
Frontooccipital fasc Sup-L	6.8 ± 0.4	6.5 ± 0.3	0.03 (0.008)	1.1 ± 0.7	1.1 ± 0.5	0.02 (0.002)

*White matter tracts where mean diffusivity (MD) and axial diffusivity (AD) were found to be significantly higher in the traumatic brain injury (TBI) group compared to healthy controls (HC), after controlling for age, sex, and time since injury at *p*-value ≤ 0.05, corrected for multiple comparisons. For each structure, the mean value of the diffusion parameters is first calculated in every subject for only those voxels where significant differences were identified, and then the mean across all subjects is calculated. The *p*-values quoted are the mean values calculated only for those voxels where significant differences were identified. The numbers in parenthesis in the *p*-value column represent *p*-value obtained in the *post hoc* analyses which was performed with only those voxels where a significant difference at *p-*value ≤0.05 was earlier found*.

*fasc, fasciculus, L, left, Post, posterior, rad, radiations, R, right, Sup, superior*.

### *Post Hoc* Analyses of the Inter-Group Differences in DTI Parameters

The *post hoc* analyses, involving only the significant voxels at *p* ≤ 0.05 level confirmed the inter-group differences in the DTI variables. The *p* values of the *post hoc* analyses are also listed in Table 3.

### Interactions

No significant interactions between the composite test scores in the three NP function categories and group variables were found, indicating that both groups exhibited similar relationships between the NP explanatory variables on the DTI parameters. Therefore, the NP-group interaction terms were excluded, and contrasts were appropriately modified for the subsequent analyses to investigate the effect of group and NP explanatory variables on the DTI parameters.

### Correlation of NP Test Scores with DTI Parameters

Continuing with the GLM design matrix with the composite test scores in the three NP function categories added as covariates along with age, sex, time since injury, education and the NART (verbal IQ), the correlation between NP test scores and DTI parameters was investigated with appropriate contrasts. In this analysis, a negative correlation was found between the composite memory score and FA in all subjects, in several tracts widespread in the entire brain. These tracts included bilateral internal and external capsules, corona radiata, cingulum, posterior thalamic radiations and superior longitudinal fasciculi. A positive correlation between composite visuomotor score and RD was also found in both groups, in a few tracts including the corpus callosum and only the right components of corona radiata, cingulum, and superior longitudinal fasciculus. Table 4 lists the tracts where a significant correlation between the DTI parameters and the composite NP test scores was found. No significant correlations between the composite NP function scores and MD or AD were found.

**Table 4:** **Tracts with significant correlations between diffusion tensor imaging parameters and composite neuropsychological scores**.

Structure	*p*-Value
Corpus callosum^a^	0.02 (0.05)
Cerebral peduncle-R	0.03
Internal capsule-R	0.03
Internal capsule-L^a^	0.03 (0.05)
External capsule-R	0.03
External capsule-L	0.03
Corona radiata-R^a^	0.03 (0.05)
Corona radiata-L	0.03
Cingulum-R^a^	0.03 (0.05)
Cingulum-L	0.03
Thalamic radiations Post-R	0.02
Thalamic radiations Post-L	0.02
Longitudinal fasciculus Sup-R^a^	0.02 (0.05)
Longitudinal fasciculus Sup-L	0.02

*White matter tracts where fractional anisotropy was found to be negatively correlated with the composite memory score, after correcting for multiple comparisons*.

*^a^The tracts where a significant positive correlation between radial diffusivity (RD) and the composite visuomotor score was also found. The *p*-values for significant positive correlations for RD are mentioned in parentheses in the second column*.

## Discussion

This study was designed to investigate the microstructural abnormalities in NAWM using DTI and to explore their potential impact on cognitive function in post-acute TBI patients compared to HC using a voxel-wise approach. Most often, TBI patients report persisting cognitive deficits and hence are not able to return to their pre-morbid daily activities, sometimes also including the inability to resume work, resulting in financial stress and increased psychological stress. The clinical assessment of this persisting cognitive deficit is challenging, especially when no gross abnormalities indicative of the cognitive or functional loss are detected on routine diagnostic imaging (e.g., structural MRI and CT). The DTI method, however, has been shown to detect micro-abnormalities and hence can be helpful in explaining the functional or cognitive deficit in sub-acute and chronic TBI with NAWM. Voxel-wise analysis of the DTI parameters and of the correlation of cognitive function with DTI parameters performed over the entire brain has the advantage of identifying partial regions or tracts with significant inter-group differences and significant correlations. Such regions may not be successfully detected *via* analyses which involve ROI selection based on *a priori* hypotheses. Partial regions or tracts with significant inter-group differences may also be masked by spatial averaging in ROI analyses.

Our neuroradiologists confirmed the absence of gross abnormalities in the central white matter of the brain in only 19 of the 40 TBI patients after reviewing their FLAIR and GRE data. These numbers suggest that some gross abnormalities due to TBI are likely to persist for lengthy time periods and may account for the changes in DTI parameters in chronic TBI, as widely quoted in the literature. It is noteworthy, however, that most of the TBI patients with NAWM that were included in our analyses had mild TBI (15 out of 19 subjects). Mild TBI is more frequent than other forms of TBI world-wide (55), and is less likely to cause gross lesions in the brain.

Our finding of widespread increase in the overall diffusivity of water molecules (as measured with MD) in the post-acute TBI subjects, while controlling for age, sex, and time since injury, robustly reaffirms the findings of previous studies (12, 13, 21, 24, 32, 33, 56, 57) and indicates the persistence of DAI in sub-acute and chronic TBI with NAWM. We also found an accompanying increase in AD in the TBI group. Many DTI studies of TBI have not analyzed AD. However, those studies that explored inter-group differences in AD also found an increase in this parameter in post-acute TBI (21, 32). The changes in AD were more widespread compared to changes in MD, and potentially significantly contributed to increase in MD.

We did not find any significant differences between the two groups in FA. Several DTI studies of TBI reported a decrease of FA in post-acute TBI; however, these studies included more acute patients, up to 1 month from the injury (22, 23, 27, 30). Some other studies with a minimum post-injury time similar to ours (2–3 months) reported variable FA results: some reported a decrease (12, 24, 26, 31, 32); one reported an increase (58); and others reported no change (9, 25, 59). One potential reason for this variability may be the presence of gross abnormalities in the brain, as some of the studies that reported decreased FA either included subjects with gross abnormalities (24, 32) or did not screen subjects for gross abnormalities (26). Furthermore, one study that found reduced FA (12) focused on TBI subjects using prescribed medicines for major depression or post-traumatic stress disorder, which constitute a known confounding factor (60).

We also did not find any significant differences between the two groups in RD; however, trends of increased RD in TBI were evident. Very often, studies of DTI in post- acute TBI have not investigated the RD parameter. A few studies involving RD reported variable findings, with increase (32) or no difference (26) observed in chronic TBI compared to HC.

A survey of the TBI literature suggests that DTI parameters change dynamically with time post-injury. One longitudinal study assessing FA values in TBI patients reported a decrease at the acute and sub-acute stage (range of time since injury = 3–55 days) when compared with controls, an increase at the chronic stage (minimum of 238 days since the first scan) in comparison with patients’ first measurements and no difference at the chronic stage when compared with controls (25). The same study also reported an increase in MD in the TBI group in comparison with controls at both time-points, and an increase in MD with time in the TBI group. Another longitudinal study reported increases in AD, MD, and FA in the acute stage, and no differences in RD; and sustained increase in MD in the chronic stage accompanied by an increase in RD, normal AD levels, and a reduction in FA compared to HC (33). These observations suggest that the absence of statistically significant differences in RD and FA in our study might be due to inclusion of both sub-acute and chronic TBI patients.

Considering these findings collectively with our results, we conclude that DAI, as manifested in elevated diffusivity, potentially persist from the acute stage to well into the chronic stage after TBI, whereas changes in FA may be more sensitive to time since injury and may not remain detectable later on in the chronic stage. The different effects shown by these parameters are interesting, and may reflect their biophysical underpinnings. Whereas MD is a linear combination of diffusivities in the three orthogonal directions and will increase if all three diffusivities are increased, FA is a measure of difference among the three diffusivities and may show no change if the three diffusivities are all modulated proportionally in the same manner. An increase in AD and MD and no difference in FA in our study, therefore, suggest a trend of increased RD values in TBI subjects. Furthermore, AD is thought to be a measure of axonal integrity (61). By extension, our results suggest that the repair of the axonal cytoskeleton, thought to result in rising AD values during recovery (24), happens before the repair of axonal membranes or re-myelination of axons, both of which are thought to restrict perpendicular diffusion and to help normalize increased RD values (62).

Persisting elevated MD values in sub-acute and chronic TBI, encompassing association (e.g., external capsule), projection (e.g., corona radiata, thalamic radiation), and commissural (e.g., corpus callosum) fibers, despite overall NAWM, suggest that microstructural damage or pathological change remains a salient feature of chronic TBI. The significant changes in MD which represent a cumulative effect of AD and RD values are of primary importance, possibly suggesting that MD can be used for assessing the burden of microstructural damage in sub-acute and chronic TBI characterized with NAWM. Our *post hoc* analyses also confirmed these differences.

Our investigation of the relationship between NP function and DTI parameters primarily revealed effects involving FA and RD, and not AD and MD. The absence of interaction effects between NP test scores and DTI parameters implies that positive and negative correlations hold similarly in all subjects regardless of the group status. A significant negative correlation was found between the composite memory score and FA in several white matter tracts. Notably, the negative correlation was found in the structures of the corpus callosum, bilateral external capsule, bilateral posterior thalamic radiations, and bilateral superior longitudinal fasciculi, which constitute either the commissural, projection, or long-range association fibers. Working and short-term memory is known to be associated with various regions in the frontal, parietal, and anterior-cingulate cortices and basal ganglia (63). The white matter tracts involved in memory are the relatively short-length or U-association fibers connecting these cortical regions. The major white matter tracts that are known to be involved in memory include the fornix (64, 65), which projects to the cingulate cortex, and the internal capsule (66), which connects the cortex with basal ganglia. Furthermore, the DTI literature suggests a positive correlation of FA with function, indicating higher FA (and hence intact structure of axons, with no disruption in myelin or axonal membranes) in subjects with better performance on memory (67) as well as other cognitive tasks (68, 69). Interestingly, our results show a negative correlation of FA in the white tracts which are not directly involved in memory. Keeping in mind the evidence of widespread DAI in the brain, we explain this finding by suggesting that whereas FA increases within the network of interest are correlated with improved performance, the negative correlation of FA in another network or some sub-network might indicate hyper-function resulting in less effective mental processing and thus a decrement in behavioral performance.

We also found a significant positive correlation between RD and visuomotor performance in the corpus callosum and right aspects of corona radiata, cingulum, and superior longitudinal fasciculus. This means that poor visuomotor coordination (higher scores on the Pegboard task) were associated with elevated RD values. Each of the structures where a positive correlation of RD with visuomotor task performance is found is known to be involved in the visual or motor tasks: the corona radiata are associated with the corticospinal tract, the superior longitudinal fasciculi are involved in integrating the motor and decision-making centers with ipsilateral visual centers (70), and the posterior aspect of cingulum is involved in visual skills. Although other studies have reported tracing of superior longitudinal fasciculi associated with investigation of language networks (71), our study is the first to report the involvement of this structure in visuomotor skills using DTI parameters.

Our study has some limitations, which include the collection of DTI data in only 12 diffusion-weighted directions with coarse resolution, the small sample size, and the inclusion of subjects with all TBI severities. Another limitation is that we did not use susceptibility-weighted imaging (SWI) to detect microstructural abnormalities in the NAWM because the protocol was not available on the MRI scanner at the time of data collection. As SWI is known to be superior to GRE in detecting micro-hemorrhages (72), it can be argued that the differences between the two groups might be influenced by undetected micro-hemorrhages in NAWM, which were not visible on GRE. However, the inter-group differences that were found are well supported by earlier studies (12, 24, 32, 73). The similarity of the effect of NP performance on the DTI parameters across both groups and the lack of interaction between NP performance and group variable also indirectly supports that no gross abnormalities were present in the white matter of subjects with TBI.

### Conclusion and Future Directions

Overall, this pilot study shows DTI to be helpful in evaluating the microstructural abnormalities in sub-acute and chronic TBI patients even when there are no apparent abnormalities in the central white matter. Elevated diffusivity values appear to persist well into the chronic stage after TBI, and hence MD can be used to assess persisting microstructural damage in chronic TBI. No clear trend for FA values in post-acute TBI is visible. The study also indicates the potential use of DTI in assessing neurocognitive functions in both TBI patients and HC. The authors recommend larger and longitudinal studies for each of mild, moderate, and severe TBI, to establish the use of DTI parameters as biomarkers of DAI and neurocognitive function in patients with NAWM.

## Ethics Statement

Research Ethics Board, St. Michael’s Hospital, Toronto, ON, Canada. M5B 1W8. Telephone: 416-864-6060 extension: 2557. REB # 11-282 (Strategic Teams in Applied Injury Research Protocol). Informed consent was obtained from the participants on a paper form, after a research team member explained the objectives and methods of the study to them.

## Author Contributions

EH and EC performed the neuroimaging analyses with help from CL and OC. NP provided input for TBSS analyses. RJ helped with the statistical analyses. MC and AB were involved in study design. SZ assisted with patient recruitment and research coordination. EH, EC, NP, and MC wrote the manuscript. All authors participated in discussing the results and editing the manuscript.

## Conflict of Interest Statement

The authors declare that the research was conducted in the absence of any commercial or financial relationships that could be construed as a potential conflict of interest. The reviewer RF and handling Editor declared their shared affiliation, and the handling Editor states that the process nevertheless met the standards of a fair and objective review.
